# A scoping review on the implementation of Global Observatory on Physical Activity recommendations for school children in Sub-Saharan Africa

**DOI:** 10.34172/hpp.2022.43

**Published:** 2022-12-31

**Authors:** Olusegun Olatunji Ojedoyin, Thayananthee Nadasan, Pragashnie Govender, Oladapo Michael Olagbegi

**Affiliations:** ^1^Discipline of Physiotherapy, School of Health Sciences, University of KwaZulu-Natal, Durban, South Africa; ^2^Harvard Medical Rehabilitation Hospital, Ikorodu, Lagos, Nigeria; ^3^Discipline of Occupational Therapy, School of Health Sciences, University of KwaZulu-Natal, Durban, South Africa

**Keywords:** Prevalence, Children, Sub-Saharan Africa, WHO, Childhood obesity, Policy

## Abstract

**Background:** Promoting physical activity (PA) is a critical first step in preventing and lowering the prevalence of non-communicable chronic diseases across all age groups. The Global Observatory on Physical Activity (GoPA) of the World Health Organization (WHO) suggested country-specific guidelines for promoting PA across all age categories to achieve this. However, despite an increase in obesity, there is no information on their compliance for pre-secondary school children in sub-Saharan Africa (SSA). We mapped evidence in the literature and described the available evidence on implementing GoPA recommendations for presecondary school children in SSA.

**Methods:** This scoping review included a search in PubMed, Google Scholar, Scopus, and Cochrane Library with the dates 2013–2020, using keywords and the terms (Physical activity OR exercise AND (GoPA recommendations OR Guidelines) AND ((presecondary school children) OR (primary school children) OR (basic school children) OR (children)). The most important data were tabulated.

**Results:** Twenty-three studies were identified of which ten were eligible for data extraction. Of these ten studies, 2 (20%) were conducted in Nigeria, 4 (40%) in South Africa, 2 (20%) in Ghana and 1(10%) each in Kenya and Senegal were extracted. None of these nations has a national plan or strategy to promote PA and reduce sedentary behaviors (SB).

**Conclusion:** A gap in the formulation of PA guidelines exists in SSA. Urgent action is needed for a national plan or strategy by individual country in SSA to reduce the burden of physical inactivity among school children in SSA.

## Introduction

 Unquestionably, encouraging physical activity (PA) is one of the most effective approaches to prevent and control hypertension and type 2 diabetes.^1^ Complications associated to physical inactivity have claimed millions of lives worldwide.^2-4^ The top 10 risk factors for the burden of disease in Sub-Saharan Africa (SSA), particularly non-communicable chronic diseases, were physical inactivity and low PA levels.^5^ Physical inactivity is currently prevalent at 22% in SSA, near to the global average of 27%, and is anticipated to increase over the coming decades.^6^ Despite high-income countries having the highest comparative burden of disease,^7^ low to middle-income countries have the highest percentage of physically inactive persons. Studies have shown that daily PA, even if it is only for a short period of time (less than 30 minutes), is associated with a lower mortality rate,^8^ a lower risk of heart-related conditions like stroke, diabetes, and even some types of cancer.^9^ For example, increased PA alone was responsible for a decrease in the risk of some cancers, type 2 diabetes, and cardiovascular disease, especially in adults.^10^ The “Global Observatory on Physical Activity” (GoPA) committee was established by the World Health Organization (WHO) in 2012 to achieve this goal of meeting the recommended PA for different categories of individuals.

 The responsibility of the compilation of country-specific standardised PA guidelines was given to GoPA, to understand better how countries and regions address the promotion of PA.^11^ The GoPA subsequently initiated and developed standardised country-specific PA profiles to summarise country-level data around 2013. The year 2014 to 2016 witnessed a breakthrough in information-gathering by the GoPA from 217 countries, of which 139 (64%) had complete, valid, and approved data for all the indicators checked.^12^ Prior to the formation of the GoPA, the WHO, in its quest to improve PA, had developed initial guidelines for implementation by countries, which the GoPA built upon immediately after it was constituted.^13^ The guidelines for children include the following:

###  Children aged 5–17 years 

 Children and teenagers between the ages of 5 and 17 should engage in 60 minutes or more of moderate to vigorous physical activity (MVPA) each day.^13^ More than 60 minutes of PA each day will provide extra health benefits.^13^

 The 2020 WHO recommendations include limiting children’s and teenagers’ time spent engaging in sedentary behaviors (SB), specifically time spent watching screens for leisure.^14^ The addition of SB guideline suggestions in the 2020 WHO guidelines marks a significant distinction between those from 2010 and 2020.^14^ Because PA has a favorable impact on children’s physical health, it is widely advocated.^15^ Childhood PA engagement has been linked to better cardiovascular responses in both children and adults.^15^ According to several research, PA has positive impacts on a number of health outcomes, including mental health and other quality of life in relation to health.^15-17^ In addition to its good impacts on mental health, PA has also been related to enhanced brain activity and cognition, which has a positive impact on academic performance.^17^

 Increased blood and oxygen flow to the brain, elevated levels of norepinephrine and endorphins that lower stress and improve mood, and improved cognition are just a few of the positive impacts of PA that have been noted.^18^ Regular participation in sports may enhance children’s behavior in the classroom in addition to these hypothesized physiological advantages, according to studies.^18,19^ Most of the arguments put forth have been explored in terms of long-term modifications brought on by regular PA. However, a new study has looked at the benefits of PA as changing risk factors for chronic illness. Acute, dynamic exercise may temporarily alter blood parameters, including triglyceride levels, high-density lipoprotein cholesterol levels, blood pressure, insulin resistance, and glucose regulation, according to a review by Warburton and colleagues.^19^ These alterations in blood pressure, cholesterol, and glucose regulation highlight the substantial influence that individual exercise sessions have on health status.

 Currently, promotion of PA is encouraged through a school-based approach as initiated by WHO through the Science and Technology in Childhood Obesity Policy (STOP) initiative.^20^

 Promoting PA in adulthood is better initiated from childhood, as a recent analysis has shown. A recent analysis of school-based PA programmes was undertaken as part of the STOP initiative of the WHO.^20^ It is a recommended intervention for children aged 6 to 12 years as improving PA improves physical fitness. These interventions may be cost-effective, but they must guarantee the involvement of all children, especially socially disadvantaged children who may experience more obstacles to being active outside of school. On any given weekday, around 1 billion pupils from throughout the world attend school.^21^ Children spend more time at school than anywhere else outside of their homes, making it a suitable place to provide children with opportunities for an active school day and outstanding physical education.

 However, a child should engage in at least a portion of their regular physical exercise outside school hours. Schools can contribute to this by spreading messages about healthy physical exercise to the community at large, including the parents and caregivers of the students. As a result, schools can offer and encourage PA for all kids and teenagers.^21^ Evidence suggests that accomplishing educational goals depends on the health and wellbeing of children and young people. Although the exact mechanisms are unknown, there is evidence that PA enhances cognitive functioning, including memory and planning, attention, and concentration.^22^ These factors all help students learn more effectively and succeed in school.

 Organised sport participation can also have positive psychological and social effects, such as social integration and the development of social skills.

 Several high-level documents related to sport and PA show the significance of schools as major centres of policy action.^20,21^

 Anecdotally, most schools are not known to have a particular PA guideline they follow, either from WHO, or from the National or provincial governments in SSA. The promotion of PA involves more than physical education classes for school children. Implementation of the PA recommendations by the WHO is critical for the reduction of the burden of non -communicable diseases and for optimal fitness. Therefore, this current scoping review was to establish the availability of PA guidelines for school children. The review mapped evidence on the availability and implementation of guidelines on PA promotion for school children in SSA.

## Material and Methods

 The concept of this scoping review is based on a suggestion made in Arksey and O’Malley’s study,^23,24^ which was later expanded upon by Levac and colleagues.^25^ The protocol of this study was developed and submitted elsewhere for publication. We followed the preferred reporting items for systematic reviews and meta-analyses extension for scoping reviews checklist to report this study.^24^

###  Study design

 This scoping review was conducted using the Joanna Briggs Institute approach, the Arksey and O’Malley framework and the Preferred Extension for Scoping Reviews and Reporting Items for Systematic Reviews and Meta-Analyses (PRISMA-ScR) the research questions that served as the basis for the scoping review was progressively narrowed throughout the trial to enable a more comprehensive outcome evaluation.^23-25^

###  Search strategy

 We searched four electronic databases (PubMed, Scopus, Cochrane library, and Google Scholar) with date limitation from January 2013 to December 2020 for relevant articles. The search strategy comprised of a combination of keywords such as “primary school”, “basic school”, “pre-secondary school”, “school children”, “school pupil”, “global observatory on physical activity”, “GoPA recommendations”, “physical activity”, “aerobic exercise”, “anaerobic exercise”, “sedentary behaviour”, “policy for physical activity”, “guideline for physical activity”, “school-based physical education”, “partnerships for physical activity” “sub-Saharan Africa”, “SSA”, Boolean terms (“AND”/“OR”) to separate keywords, and Medical Subject Heading terms where applicable. Study design limitations were removed during the database search. However, the search language was limited to English where possible.

###  Eligibility criteria and selection of evidence sources

 This scoping review articles were limited to sub- Saharan African only, articles that focussed on primary schools, articles in English Language only, articles that involved PA (aerobic exercises, anaerobic exercises, strengthening and flexibility exercises), and articles that reported findings on the implementation of the GoPA recommendations. Peer-reviewed articles and literature (policies, guidelines, and plans/strategies) were also included. Also, all interventional, qualitative, observational, and mixed methods study were included. All the articles were only those published between 2013 and 2020.However, articles that focussed on secondary schools and institutions of higher learning, and those published before 2013 and after 2020 were excluded from this study.

###  Identifying the research questions

 The main question for this scoping review was: *“To date, what evidence exists on the implementation of the GoPA recommendations for school children in SSA?”* The Population, Concept and Context (PCC) framework^26^ was used to determine the eligibility of this review question (Table 1). The sub-review questions included are as follows:

1. What evidence exists on national plans or strategies aimed to improve PA and reduce sedentary behaviour patterns for pre-secondary school children in SSA?
2. What evidence exists on school-based physical education and school-based sports for pupils in SSA?
3. What is the evidence on PA and sedentary behaviour of pupils in SSA’s primary schools?
4. What evidence exists on the GoPA recommendation for multisectoral partnerships for the implementation of PA strategies in SSA?

**Table 1:** PCC used to determine the eligibility of this review main research question

**PCC framework**	**Definition**
Population	Primary schools/children: These included all pre-secondary schools or children at the basic level of education except kindergarten and crèche.
Concept	GoPA recommendations: These included recommendations on PA and sedentary behaviour in schools, national policies/strategies, school-based physical education, and multisectoral partnerships for implementation.
Context	SSA countries: These included all African countries except Morocco, Egypt, Libya, Algeria, Tunisia, Djibouti, Sudan, and Somalia.^27^

*Note*. PCC:Population, Concept and Context; PA: physical activity; SSA: sub-Saharan Africa; GoPA: Global Observatory on Physical Activity.

###  Study selection

**Figure 1:**
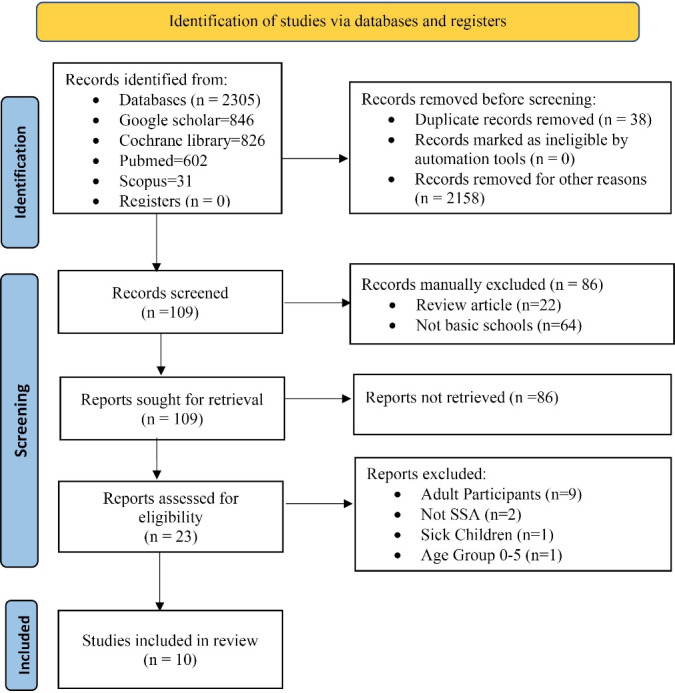
The diagram of the study based on PRISMA checklist^28^

 The article selection process consists of four steps: identification of relevant articles, screening of the title and abstract screening, assessing eligibility based on full-text screening, and finally, inclusion of the article for the review.^24,25,27^ The number of retrieved articles at each stage is illustrated in Figure 1.

###  Charting data

 A data charting form was utilized to extract and summarize the data from individual articles included in this scoping review, as advised by the work of Arksey and O’Malley and others.^23-25,27^ The information that was taken from the article includes the title, author(s), journal, and year of publication in addition to details about the study’s methodology, design, location, and participants’ characteristics.

###  Summarising and reporting the results

 We obtained 2305 articles from the four databases during the initial search. About 121 articles qualified for abstract screening, after the exclusion of duplicates, about 109 different articles remained. Applying our inclusion criteria, 23 different articles qualified for full text screening with 88.5% agreement between the two independent reviewers which constituted a substantial consensus (Cohen’s kappa = 0.90, calculated probability of < 0.05). With further application of inclusion criteria, the articles left for final review were 10. Thematic analysis using deductive reasoning was employed to collate all relevant themes and sub-themes relating to this study’s research questions. The themes were structured around the following: availability of national plans/strategies for improving PA and reduce SB, school-based physical education and school-based sports for pupils, evidence of PA and/sedentary behaviour of pupils in primary schools, and evidence of GoPA recommendations and multisectoral collaboration.

 In order to provide a thorough summary of the findings from the reviewed articles, as Arksey and O’Malley and others described the final stage of scoping reviews. In this case, the results of the studies pertaining to the GoPA guidelines for the promotion of PA among children were the main focus.^23-25,27^

## Results

###  Selection

 After eliminating duplicates, 23 articles were available for review after the literature search produced 2305 results. The systematic scoping review included ten articlesfrom the 23 full texts considered eligible (Figure 1).^29-39^

###  Study characteristics

 The systematic search strategy as demonstrated in Figure 1 yielded 10 articles which were considered eligible for this review. Table 2 illustrates the details of the 10 articles used for the final analysis and discussion, while Table 3 illustrates each article, key findings, and implications. All the studies were carried out in sub- Saharan Africa. The studies can be categorised into four themes based on deductive reasoning with regard to research questions. These are, namely, *PA and SB of pupils in SSA, Availability of national plans/strategies for improving PA, School only- based physical education and sports, and multisectoral collaborations for GoPA*.Amongthe selected studies, four were carried out in South Africa,^24-26,28^ two in Nigeria,^34,35^ two in Ghana,^36,37^ and one each in Senegal,^38^ and Kenya.^39^ Seven of the articles eligible for this review fall under the first theme multisectoral collaboration on GoPA. They were country specific report cards for South Africa, Nigeria, Ghana, and Kenya.^29,33-37,39^

**Table 2:** Characteristics of the articles included

**Authors, Year**	**Age of the participants**	**Study design**	**Setting**	**GoPA/ Strategy**	**Country of study**
De Villiers et al, 2015^31^	6 and 13 years	Mixed method	School setting	Nutritional advice/PA	South Africa
Ocansey et al, 2016^37^	Children and youth	Descriptive	Country report card	Report card	Ghana
Uys et al, 2016^30^	5-13 years	Prospective interventional	School setting	School based PE and Sports	South Africa
Adeniyi et al, 2016 ^34^	Children and youth	Descriptive	Country report card	Report cards	Nigeria
Akinroye et al, 2014 ^35^	Children and youth	Descriptive	Country report card	Report card	Nigeria
Diouf et al, 2016 ^38^	8-11 years	Cross-sectional	School setting	GoPA strategy	Senegal
Ocansey et al, 2014 ^36^	Children and youth	Descriptive	Report card	Report cards	Ghana
Uys et al, 2016 (Report)^29^	Children and youth	Descriptive	Report card	Report cards	South Africa
Draper et al, 2014^32^	Children and youth	Descriptive	Report card	Report cards	South Africa
Onywera et al, 2016^39^	Children and youth	Descriptive	Report card	Report cards	Kenya

*Note*. PA: physical activity; GoPA: Global Observatory on Physical Activity.

**Table 3:** Key findings and Implications

**Authors, Year**	**Key findings**	**Implication**
**Evidence of GoPA recommendations/multisectoral partnerships**		
Draper et al, 2014^3,2^	50% or more of children and youth were not meeting recommended levels of PA.	It is best to avoid fast meals, soft drinks, and sedentary behavior. There should be a rush on national PA policies.
Adeniyi et al, 2016^3,4^	Children and youth in Nigeria appeared to have lower total PA levels than in the previous assessment. Children and youth in Nigeria still engage in high levels of sedentary behavior and increasing obesity.	They need to be encouraged to participate in PA through school-based initiatives and PA policy.
Akinroye et al, 2014^35^	Children and youth in Nigeria had modest amounts of PA, but high levels of sedentary behavior. Policy and practice of healthy living among Nigerian children and youth can be improved with the adoption of national recommendations for PA and sedentary behaviors.	High sedentary behaviours will lead to increased cardiometabolic diseases among children. PA policy should be introduced.
Uys et al, 2016^29^	Over 50% of children's PA levels overall met recommendations. Participation in organized sports is likewise strong (almost 50%). Government policies continue to be optimistic. Children's screen time and sedentary behavior were big concerns. There is an increase in obesity.	There is better government support in South Africa for PA promotion. Although, overweight is increasing because of increased sedentary behaviour among children.
Ocansey et al, 2014^36^	A third of Ghanaian children and youth participate in insufficient PA.	School based -education and after-school sports policies and programs are needed.
Ocansey et al, 2016^37^	Overall, PA received a low score.	The adoption of national guidelines can help children's PA overall.
Onywera et al, 2016^39^	Although total PA is less than 50%, 60% of school-aged children commute actively to and from school. Sporting events organisation are poor.	To introduce PHE in schools and National PA policy should be introduced.
**Schools based physical education and school-based sports for pupils**		
Uys et al, 2016^30^	There was no general increase in physical fitness. In the intervention group, the sit-ups score increased significantly (*P* < 0.05). On the factors of PA behavior, no overall intervention effects were discovered. Both the intervention (*P* = 0.005) and control (P < 0.001) groups showed an increase in knowledge.	The lack of a distinct intervention effect on fitness levels and PA-related KAB Questionnaire results in a more favorable outcome for objective assessment employing intervention.
De Villiers et al, 2015^31^	The 3-year intervention produced the best outcomes in the action area for school food and nutrition, with 55.5% of the specified actions being carried out. The staff health activity areas of 25.9% and 20%, as well as the school's athletic and PA settings, respectively, and 54.2% of the actions in the chronic illness and diabetes awareness area were completed. According to educators, the poor implementation was due to a lack of parental involvement, time, and resources, as well as the socioeconomic and physical circumstances at schools.	The implementation of the Health Kick initiative did not proceed as smoothly as anticipated. Future interventions must take action to ensure sufficient support from the schools, family and from the government.
**National plans/ strategies aimed to improve PA and reduce SB**		
None	None	None
**Evidence of PA/SB of pupils in SSA**		
Diouf et al, 2016^38^	54.8% (n = 23) of the students completed 60 minutes of MVPA per day. Girls' MVPA reduced as their body fat percentage increased.	Reducing sedentary behaviour should be the major focus.

*Note*. PA: physical activity; SSA: sub-Saharan Africa; GoPA: Global Observatory on Physical Activity; KAB: Knowledge, Attitude, and Behavior; SB: sedentary behaviors; MVPA, moderate to vigorous physical activity; PHE: physical and health education.

 The country-specific report cards were essentially a product of extraction of secondary data from archives and literature to provide a data bank for the relevant countries as recommended by the GoPA.^11^ GoPA released the first batch of “Country Cards,” or PA profiles, for 139 nations in 2015, using data up to 2013. Using data from 217 countries up to 2019, GoPA produced the Second Set of Country Cards in January 2021, after 6 years and utilising standardised methodologies. More than 150 of these 217 nations are represented locally by members of the GoPA Network. The country-specific report cards were special review, because these were reviews advised by GoPA as a form of multisectoral collaboration to create a data bank for each country to assist for PA promotion hence the inclusion.

 The second theme,*School-based physical education and sports*has two studies both from South Africa.^30,31^

 The third theme, *evidence of PA, and SB of pupils in SSA* had a study.^38^ While the fourth theme, *National PA plans/strategies* had no study.

###  Brief descriptions of studies included

 A cross-sectional study of schoolchildren in Dakar, Senegal, was carried out by Diouf et al. According to the findings, girls’ MVPA reduced as their body fatness increased. Although the two techniques used to measure low and moderate PA levels are inconsistent. The PAQ-C (Physical Activity Questionnaire for Older Children) is a simple routine approach that must be validated for African children because different activities were not tailored for genuine activities in Senegalese children. Overall, there was no set policy for Senegalese kids.^38^

 However, De Villiers et al in their study designed the Health Kick intervention’s behavioural outcomes, which promote a balanced diet and regular exercise, using the intervention mapping method. This intervention was designed for South African schools. It is very similar to a PA guideline for schools if properly implemented.^26^ Impact of a South African School-based Intervention, Health Kick, on Fitness Correlates was examined by Uys et al in 2016.^30^

 According to Ocansey and colleagues, Ghana’s 2014 and 2016 report cards (RC) were created by the Active Healthy Kids Ghana Organization using the data on children’s and youth’s PA levels that was available at the time. By highlighting areas where Ghana is making progress as a nation and emphasizing areas that require more attention/action, these report cards were designed to raise awareness and sensitivity regarding concerns surrounding PA and SB of children and youth. This method of assessing children’s and adolescents’ PA levels is reliable and durable. The findings of the 2016 Ghana RC mirror those of the 2014 Ghana RC.^36,37^ The levels of PA did not change. They both claimed that fewer than 40% of Ghanaian children had adequate levels of PA.

 Adeniyi et al and Akinroye et al report cards on Nigeria for 2016 and 2014 respectively were both based on data from the literature and archives on children’s and youths’ PA levels.^34,35^ Compared to the report from 2014, the 2016 report card revealed a decrease in the overall levels of PA. According to Akinroye and colleagues in the 2014 report card, Nigerian children and youth had moderate PA levels but high levels of sedentary behaviour. They suggested that national recommendations for PA and sedentary behaviours be developed to effectively guide practice and policy about healthy living among Nigerian children and youth.^34,35^

 More than 50% of children in South Africa, according to the two studies that served as report cards for children and youth, adhere to the PA recommendations.^29,33^

 In their study, Onywera and colleagues^39^ found that Kenyan youth are becoming more sedentary than their counterparts in rural areas, particularly those from metropolitan areas. Children in rural areas were more physically active than those in urban areas, according to step-count data.^40^ Around 72% of these urban and rural children were categorized as meeting the minimum recommended 60 minutes daily MVPA for aged 5-17 years.^14^

## Discussion

 The current scoping review focused on PA guidelines for primary school children for which the literature was collected systematically and was not limited to randomised controlled trials or dependent on quality levels providing a broader overview of the existing literature and description of the used methodologies. In all the articles searched, no country has at least a guideline for PA for primary school years in compliance with the GoPA recommendations for different categories of individuals. It is also crucial to point out that studies from South Africa was included in two of the four areas that were examined in line with the research questions: evidence of GoPA recommendations/multisectoral collaborations, school-based physical education, and student sports. Nigeria featured in one of the themes, which is GoPA Recommendations/Multisectoral Partnerships. Ghana and Kenya featured under the evidence of GoPA Recommendations/Multisectoral Partnerships, and finally Senegal featured under the evidence of PA/SB in pupils. Other countries in SSA are not featured at all in any of the themes. The implication is that just five countries can be said to be addressing the PA promotion among this cohort of member countries as advised by GoPAin SSA.^11^ Also, South Africa is the only country in SSA that is addressing physical inactivity in children through a two-pronged approach of GoPA multisectoral partnerships and evidence of school-based studies on physical education and sports activities in children. Still, none of these countries has a national plan or strategy to promote PA and reduce SB.

 This systematic scoping review provided evidenced based knowledge on the non-availability of PA guidelines for school children in SSA. The findings revealed a lack of proper PA guideline for basic school children in all the countries that make up SSA. Our results demonstrated that there is more to be done by policymakers in all the affected countries. Additionally, our results presented a scarcity of studies focusing on other age groups. This study observation highlights a gap of knowledge on the non- availability of PA guidelines in SSA, which may have a potential adverse effect on Populace’s health.

 It is important to note that, as indicated in Table 3, our study has been able to address our research questions.

 However, this study was able to identify a gap in all of the countries’ national goals and plans to boost PA and cut back on SB. Introduced as a component of the WHO’s STOP initiative is the school-based PA programs which can be used to address this.^20^

###  Strengths of the evidence-based interventions to promote PA through schools

 No single action can guarantee that all children or students in a particular school engage in the recommended amounts of PA. The best strategy is to maximise the Health Promoting Schools initiative that incorporates a whole-school approach to providing physical exercise opportunities in classrooms.^20,41-44^

 It has been demonstrated that six domains work well to assist the promotion of PA using a whole-school strategy as incorporated in the Health Promoting Schools initiative. Below is a discussion of the six domains.^20^

####  a. Provide PA through quality physical education

 High-quality physical education should focus on teaching physical competence and confidence, sport and movement skills, and health awareness. Physical education, which guarantees access to and appreciation for physical activities that improve health,^21^ can possibly be beneficial for the majority of children. The opportunity to increase PA during the school day is provided by hysical education. It can provide kids with the chance to master a variety of physical skills so they can enjoy being physically active. If children develop confidence and competence in PA, they will be more inclined to be active on their own time.^45-50^

####  b. Put in place measures to promote active transportation to and from school

 Walking, cycling, and other forms of active transportation are alternatives to motorised transport (such as vehicles, motorcycles). Public transport may also be included because getting to the bus, train, or other means of transportation typically necessitates exerting physical effort.^20^ Active transport to and from school offers most pupils the best opportunity to enhance their daily habitual PA when it is safe to do so.^45^

####  c. Provide active before- and after-school programmes

 Before and after-school activities, commonly referred to as out-of-school-hours (OSH) activities,^46^ are scheduled opportunities for PA that take place outside of the classroom. Depending on the local conditions, OSH exercises may be designed and delivered at the school by staff, volunteer peer leaders, parents, or carers, or in the neighborhood by externally funded, non-profit or commercial organizations. They should be available to all students, either for free or at a cost that will allow everyone to benefit from them.^20^

####  d. Provide PA opportunities during recess and recreation time

 Due to the opportunity for PA, all pre-secondary school grade levels should have access to recess and recreation time. By providing PA opportunities during breaks and free periods, schools can improve their student life by reducing inactivity,^47^ SB, boredom, and poor behavior. Schools should provide safe, accessible indoor and outdoor spaces for kids and teens to participate in PA during these times.

####  e. Embed active classrooms in school curricula

 For in-class PA, any period during the school day is acceptable. For example, by interjecting during courses with quick (3–5 minutes). Incorporating PA into the delivery of academic content (for instance, counting jumps as part of basic mathematics or counting steps taken to estimate distance), and rearranging the classroom to increase PA or decrease SB (for example, by introducing standing desks or activity equipment or by using outdoor spaces).^48^

####  f. Make sure inclusive PA strategies are used for kids with special needs.

 The International Charter of Physical Education, Physical Activity, and Sport’s General Conference notes that “the practice of physical education, exercise, and sports are universally recognised as fundamental rights.^49^ From the physically challenged to the physically brilliant. Schools are accountable for delivering a curriculum for all students that matches their unique needs. The promotion of universal primary school completion, cost-effectiveness, and the eradication of discrimination are all aided by including children with disabilities or chronic ailments in regular school activities.^49,50^

###  Failures of school PA policy in Sub-Saharan Africa

 The main causes of the failures of policies in SSA have been identified as a lack of political will, a lack of technical expertise, ethnicity, and finally financial and professional support by major stakeholders in the health and education sector as mentioned in a study by Onimisi et al in their study titled ‘*Factors affecting effective policy implementation in NIGERIA: Focus on federal character principle.*’^51^

 Only English-language publications that were published in peer-reviewed journals were considered in this review. This may have reduced the evidence by excluding papers that could have been appropriate for inclusion in this evaluation from the francophone nations of SSA. Second, as recommended by GoPA, seven papers in this study were report cards, essentially reviews of secondary literature. Because of this, it qualified as a unique review and was incorporated into the research. The report cards were the first globally distributed, nation-by-nation cards to demonstrate how each nation reacted to the GoPA’s call to action.

## Conclusion

 The scant number of discovered papers highlights the paucity of research on PA recommendations for all SSA population categories. Additionally, this review has revealed that roughly 90% of the SSA nations still do little or nothing to promote PA among pre-secondary school children.

 This study shows no evidence of a PA guideline as recommended by GoPA for Nigeria and other SSAn countries. Just around 10% of the total countries in SSA are moving in positive direction, although this progress might be slow. Promotion of PA through the STOP initiative should be encouraged. Six domains are effective to assist in the promotion of PA using a whole school strategy as discussed above even when there is no existing PA policy for school children. Additionally, more future studies should be conducted at different countries within SSA to develop PA guidelines for different age categories to forestall an increase in metabolic conditions like obesity and even prevent early onset of chronic non-communicable chronic diseases among children.

## Acknowledgements

 We are grateful to Dr D Kuupiel, College of Health Sciences, University of KwaZulu-Natal, Durban, South Africa, for his expert guidance in the development of this scoping review.

## Author Contributions

**Conceptualization:** Olusegun Olatunji Ojedoyin, Thayananthee Nadasan, Pragashnie Govender.

**Data curation:** Olusegun Olatunji Ojedoyin, Thayananthee Nadasan, Pragashnie Govender.

**Formal Analysis:** Olusegun Olatunji Ojedoyin, Pragashnie Govender.

**Funding Acquisition:** Thayananthee Nadasan, Pragashnie Govender, Oladapo Michael Olagbegi.

**Investigation:** Olusegun Olatunji Ojedoyin.

**Methodology: **Olusegun Olatunji Ojedoyin.

**Project Administration: **Olusegun Olatunji Ojedoyin, Thayananthee Nadasan, Pragashnie Govender.

**Resources: **Olusegun Olatunji Ojedoyin.

**Supervision: **Thayananthee Nadasan, Pragashnie Govender, Oladapo Michael Olagbegi.

**Validation:** Olusegun Olatunji Ojedoyin, Thayananthee Nadasan, Pragashnie Govender, Oladapo Michael Olagbegi.

**Visualization:** Olusegun Olatunji Ojedoyin, Thayananthee Nadasan, Pragashnie Govender, Oladapo Michael Olagbegi.

**Writing – original draft: **Olusegun Olatunji Ojedoyin.

**Writing – review & editing:** Thayananthee Nadasan, Pragashnie Govender.

## Funding

 The first author has received tuition remission as part of PhD studies and operational funds from the College of Health Sciences, University of KwaZulu- Natal, Durban, South Africa.

## Ethical Approval

 Not applicable because secondary data were used.

## Competing Interests

 The authors declare that they have no competing interests.
